# Supplementary material for: Demographic and Clinical Factors Associated With SARS-CoV-2 Spike 1 Antibody Response Among Vaccinated US Adults: the C4R Study
Source: Nat Commun. 2024 Feb 19;15:1492. doi: 10.1038/s41467-024-45468-9 (PMC10876680; doi:10.1038/s41467-024-45468-9)
Supplement: Supplementary file 1 — Supplementary Information [file 41467_2024_45468_MOESM1_ESM.pdf]

Demographic and Clinical Factors Associated With SARS-CoV-2 Spike 1 Antibody Response Among Vaccinated US Adults: the C4R Study

SUPPLEMENTARY INFORMATION

Supplementary Methods: Description of C4R Cohorts

Atherosclerosis Risk in Communities (ARIC): The ARIC study began in the mid 1980s with initial aims for its cohort component being to describe the presence of subclinical atherosclerosis (mainly via carotid ultrasound), the progression of atherosclerosis to clinical cardiovascular disease (CVD), and the association of novel risk factors with CVD. ARIC recruited its cohort of 15,792 men and women aged 45-64 in 1987-89 from four communities: Forsyth County, NC; Jackson, MS; suburban Minneapolis, MN; and Washington County, MD. The investigators used probability sampling to obtain a community wide sample, exclusively sampling African Americans in Jackson and oversampling African Americans in Forsyth County. ARIC conducted a baseline examination of cohort participants and up to seven subsequent examinations; performed annual or semi-annual telephone follow-up interviews; and throughout has identified and validated incident CVD and other outcomes, particularly cognitive decline in recent years.

Coronary Artery Risk Development in Young Adults (CARDIA): CARDIA is a study examining the development and determinants of clinical and subclinical CVD and their risk factors. It began in 1985-1986 with a cohort of 5115 Black and White men and women aged 18-30 years. The participants were selected so that there would be approximately the same number of people in subgroups of race (Black and White), gender (female and male), education (high school or less and more than high school) and age (18-24 and 25-30 years) in each of 4 field centers: Birmingham, AL; Chicago, IL; Minneapolis, MN; and Oakland, CA. These same participants were asked to participate in follow-up examinations during 1987-1988 (Year 2), 1990-1991 (Year 5), 1992-1993 (Year 7), 1995-1996 (Year 10), 2000-2001 (Year 15), 2005-2006 (Year 20), 2010-2011 (Year 25), 2015-2016 (Year 30), and 2020-2022 (Year 35). A majority of the group has been examined at each of the follow-up examinations (91%, 86%, 81%, 79%, 74%, 72%, 72%, 71%, and 67% [despite the impact of the COVID-19 pandemic on Year 35], respectively). While the specific aims of each examination have varied, data have been collected on a variety of factors believed to be related to heart disease. These include conditions with clear links to heart disease such as blood pressure, cholesterol and other lipids, and glucose. Data have also been collected on physical measurements such as weight and body composition as well as lifestyle factors such as dietary and exercise patterns, substance use (tobacco and alcohol), behavioral and psychological variables, medical and family history, and other chemistries (e.g., insulin). In addition, subclinical atherosclerosis has been measured via echocardiography during Years 5, 10, 25, and 30, a chest CT scan during Years 15, 20, 25, and 35, an abdominal CT scan during Years 25 and 35, and carotid ultrasound during Year 20. A brain MRI was performed on a subset of participants at Years 25, 30, and 35. The CARDIA cohort, born between 1955 and 1968, has been influenced substantially by the obesity epidemic at ages younger than participants in other established NHLBI cohorts. Further investigation of the mechanisms linking obesity to derangements in cardiovascular structure and function and the etiology of clinical events promises to generate important new knowledge to inform health promotion and disease prevention efforts.

Genetic Epidemiology of COPD (COPDGene): COPDGene is a non-interventional, multicenter, longitudinal, case-control study at 21 US sites of smokers with a ≥10 pack-year history with and without COPD and healthy never smokers. The goal was to characterize disease-related phenotypes and explore associations with susceptibility genes. COPDGene research participants were extensively phenotyped with the use of comprehensive symptom and comorbidity questionnaires, spirometry, chest CT scans, and genetic and biomarker profiling. The study enrolled 10,198 participants. COPDGene has had 3 exams that include spirometry, diffusing capacity, lung CT scans and other measures; its current exam is ongoing. COPDGene examines the influence of age, sex, and race on the natural history of COPD, and the impact of comorbid conditions, chronic bronchitis, exacerbations, and asthma/COPD overlap.

Framingham Heart Study (FHS): FHS was initiated in 1948. Researchers recruited 5,209 men and women between the ages of 30 and 62 from the town of Framingham, Massachusetts, and began the first round of extensive physical examinations and lifestyle interviews that they would later analyze for common patterns related to CVD development. Since 1948, the subjects have returned to the study every two years for an examination consisting of a detailed medical history, physical examination, and laboratory tests, and in 1971, the study enrolled a second-generation cohort – 5,124 of the original participants' adult children and their spouses – to participate in similar examinations. The second examination of the Offspring cohort occurred eight years after the first examination, and subsequent examinations have occurred approximately every four years thereafter. In April 2002 the Study entered a new phase: the enrollment of a third generation of participants, the grandchildren of the original cohort. The first examination of the Third Generation Study was completed in July 2005 and involved 4,095 participants. Thus, the FHS has evolved into a prospective, community-based, three generation family study. In addition to research studies focused on risk factors, subclinical CVD and clinically apparent CVD, Framingham investigators have also collaborated with leading researchers from around the country and throughout the world on projects involving some of the major chronic illnesses in men and women, including dementia, osteoporosis and arthritis, nutritional deficiencies, eye diseases, hearing disorders, and chronic obstructive lung disease.

Hispanic Community Health Study/Study of Latinos (HCHS/SOL): HCHS/SOL is an ongoing population based prospective cohort study of 16,415 community dwelling Hispanic/Latino adults aged 18-74 years at baseline, recruited from four urban field centers with large populations of Hispanics/ Latinos (Bronx, NY; Chicago, IL; Miami, FL; and San Diego, CA). A two-stage area probability sample of households was selected, with stratification and over-sampling at each stage to ensure a diverse and representative sample.[39] Participants self-identified as Hispanic/Latino and of Cuban, Dominican, Mexican, Puerto Rican, Central American, South American, or other/more than one heritage. Study participants underwent an extensive clinic exam and assessments to determine baseline risk factors (2008-2011),[40] and annual telephone follow-up interviews for ascertainment of cardiovascular and pulmonary events. A second clinic visit was conducted in 2014-2017, and a third clinic visit is now in process (2020-2022). The overall retention rate as of December 2019 was 81.9%. The primary goals of the HCHS/SOL are to describe: (1) the prevalence and incidence of cardiovascular, pulmonary, and other major chronic conditions (2) the risk and/or protective factors associated with these conditions; and (3) the relationships between the initial sociodemographic and health profiles and future health events in the target population. The study to date has revealed a high prevalence of cardiovascular risk factors, with significant variability by Hispanic/Latino heritage and sociodemographic factors such as income and time in the United States.[41]

Jackson Heart Study (JHS): The JHS is a community-based cohort study evaluating risk factors for cardiovascular and related diseases among adult African Americans residing in the three counties (Hinds, Madison, and Rankin) that make up the Jackson, Mississippi metropolitan area. Data and biologic materials have been collected from 5,306 participants, including a nested family cohort of 1,498 members of 264 families. The age at enrollment for the unrelated cohort was 35-84 years; the family cohort included related individuals >21 years old. Participants have provided extensive medical and psychosocial histories and had an array of physical and biochemical measurements and diagnostic procedures during a baseline examination (2000-2004) and two follow-up examinations (2005-2008 and 2009-2012). Samples for genomic DNA were collected during the first two examinations. Annual follow-up interviews and cohort surveillance of cardiovascular events and mortality are continuing and a fourth examination is in progress.

Mediators of Atherosclerosis in South Asians Living in America (MASALA) study[42,43]: South Asians comprise almost one-quarter of the world's population and are the second fastest growing ethnic group in the US. The MASALA Study is a prospective cohort of South Asians called the MASALA study, which

is closely tied to the Multi-Ethnic Study of Atherosclerosis (MESA), for valid cross-ethnic comparisons.[43] MASALA enrolled 906 South Asians in 2010-2013 and then added a new wave of 258 South Asian participants from 2017-2018, for a full cohort size of 1,164.[42] The original MASALA cohort has been followed for approximately 8.5 years, and completed a second clinical exam in early 2018. A third MASALA clinical exam is planned for 2022-2024. 75 papers have been published from MASALA to date, and the findings clearly show that the US South Asian population has a distinct phenotype compared to the other four race/ethnic groups studied in MESA. Major findings have included a higher prevalence of diabetes, ectopic adiposity and coronary artery calcium compared to MESA. The MASALA study findings have influenced guidelines for diabetes screening, lipid management, and raised awareness of South Asian CVD risk. MASALA is filling a large gap in scientific knowledge about CVD in a large, growing Asian American subgroup.

Multi-Ethnic Study of Atherosclerosis (MESA): MESA is a study of the characteristics of subclinical CVD (disease detected non-invasively before it has produced clinical signs and symptoms) and the risk factors that predict progression to clinically overt cardiovascular disease or progression of the subclinical disease. MESA researchers study a diverse, population-based sample of 6,814 asymptomatic men and women aged 45-84. Thirty-eight percent of the recruited participants are white, 28 percent African-American, 22 percent Hispanic, and 12 percent Asian, predominantly of Chinese descent. Participants were recruited from six field centers across the United States: Wake Forest University, Columbia University, Johns Hopkins University, University of Minnesota, Northwestern University and University of California – Los Angeles. Each participant received an extensive physical exam and determination of coronary calcification, ventricular mass and function, flow-mediated endothelial vasodilation, carotid intimal-medial wall thickness and presence of echogenic lucencies in the carotid artery, lower extremity vascular insufficiency, arterial wave forms, electrocardiographic (ECG) measures, standard coronary risk factors, sociodemographic factors, lifestyle factors, and psychosocial factors. Selected repetition of subclinical disease measures and risk factors at follow-up visits allows study of the progression of disease. Blood samples have been assayed for putative biochemical risk factors and stored for case-control studies. DNA has been extracted and lymphocytes cryopreserved (for possible immortalization) for study of candidate genes and possibly, genome-wide scanning, expression, and other genetic techniques. Participants are being followed for identification and characterization of cardiovascular disease events, including acute myocardial infarction and other forms of coronary heart disease (CHD), stroke, and congestive heart failure; for CVD interventions; and for mortality. In addition to the six Field Centers, MESA involves a Coordinating Center, a Central Laboratory, and Central Reading Centers for Computed Tomography (CT), Magnetic Resonance Imaging (MRI), Ultrasound, and Electrocardiography (ECG). Protocol development, staff training, and pilot testing were performed in the first 18 months of the study. The first examination took place over two years, from July 2000 – July 2002. It was followed by five examination periods that were 17-20 months in length. Participants have been contacted every 9 to 12 months throughout the study to assess clinical morbidity and mortality. The MESA Lung Study enrolled 3,965 MESA participants in 2004-06 and has performed spirometry 3 times and full-lung CT scans twice, most recently in 2016-18.

Northern Manhattan Study (NOMAS): NOMAS began in 1993 as a population-based incidence and case-control study. In 1998 (cycle 2) the study evolved into a prospective cohort study of 3,298 stroke-free, tri-ethnic, community subjects followed annually to detect stroke, MI, and death. Starting in 2003 (cycle 3), subclinical measures (brain MRI & carotid ultrasound) and the first complete neuropsychological (NP) battery were collected on 1290 members (MRI cohort). The project has remained productive through subsequent cycles. As the cohort aged, the specific aims grew to include not only vascular determinants of stroke but also cognitive decline, mild cognitive impairment (MCI) and dementia. NOMAS participates in collaborative studies on genetics, stroke, MRI markers, Alzheimer Disease and neurodegenerative diseases. One of the major interests of the study has been the exploration of inflammatory and infectious contributors to stroke risk, subclinical atherosclerotic and cerebrovascular

disease, and cognitive decline. The NOMAS community cohort of 3,298 subjects was assembled from a population-based, random sample based on the following criteria: (1) resident of at least 3 months of Northern Manhattan; (2) randomly derived from a household with a telephone; (3) age 40 or older at baseline (changed to age 55 in 1998); and (4) no history of stroke. The 1,290 subjects in the MRI cohort (median age 70 at MRI; 60% women, 15% non-Hispanic White, 17% non-Hispanic Black, 66% Hispanic, 2% Other) were evaluated with a standardized brain MRI and NP battery between 2003-08. The cohort has been prospectively followed with annual telephone contacts, including the Telephone Interview for Cognitive Status (TICS), and 3 in depth neuropsychological evaluations at 5 year intervals in the MRI cohort. The aging cohort is representative of an elderly, urban, diverse community at risk for cognitive decline. A wealth of data was collected during baseline enrollment and at time of MRI and 1st NP visit, including socio-demographics, psychosocial and socioeconomic status (education, occupational attainment, insurance status), medical history, medications, risk factors, family history and other health data, behavioral/environmental factors, subclinical vascular measures, serum biomarkers (infectious burden, neuroimmune markers using a novel multiplex assay, HOMA index for insulin resistance, adiponectin, CRP, homocysteine), carotid imaging, echocardiographic imaging (LV, LA size), ambulatory BP and cardiac rhythm monitoring, brain MRI biomarkers (regional brain volumes, regional white matter lesion burden, hippocampal volumes, cortical thickness, covert infarcts, cerebral microbleeds, perivascular spaces, brain arterial diameters), and genetic markers (GWAS, ApoE4). Fasting blood was collected and stored at baseline and at MRI. Subjects had complete blood count, chemistry profile, total protein, albumin, calcium, markers of mineral metabolism (fibroblast growth factor 23, parathyroid hormone, 1,25OH and 25OH vitamin D, and phosphate), CRP, TNF receptor levels, IL-6, and serologies against some viral and bacterial pathogens. Fasting plasma levels were assayed for total and HDL cholesterol, lipoprotein (a), HDL particle size, triglycerides, lipoprotein-associated phospholipase A2, homocysteine, serum insulin levels, and adiponectin. Buffy coats and DNA were stored on 2433 subjects and ApoE4 genotype is available on the MRI cohort. We continue to follow the cohort with annual telephone contacts and a 4th NP assessment to track cognitive trajectories and adjudicate MCI and dementia. Cognitive, functional, quality of life, and social situation questions are assessed annually. The National Death Index is consulted periodically for those with unknown vital status. A surveillance system at CUIMC detects hospitalizations, ED visits, and clinical visits. Remarkably, only 3 (0.38%) subjects are lost, and 11 (1.4%) have withdrawn from active participation.

Prevent Pulmonary Fibrosis (PrePF): PrePF has been investigating the clinical, physiologic and genetic phenotypes of interstitial lung disease (ILD) by focusing on families with two or more cases of ILD and individuals with sporadic IPF. It has recruited over 1200 families with two or more cases of pulmonary fibrosis. These families with pulmonary fibrosis include 2837 individuals with probable or definite idiopathic interstitial pneumonia (IIP) and 2404 unaffected FDRs. In addition, PrePF recruited over 10,000 individuals with sporadic idiopathic pulmonary fibrosis (IPF).

REasons for Geographic and Racial Differences in Stroke (REGARDS): the REGARDS cohort is one of the nation's largest, most comprehensive population-based cohorts, its innovative home- and telephone-based data collection is nimble and cost-efficient. REGARDS centrally recruited and initially examined 30,239 non-Hispanic Black and White men and women aged ≥45 years in 2003-7 by telephone and in participant homes across the 48 contiguous US states (62% of US counties). Over 17 years, REGARDS has collected follow-up data by computer-assisted telephone interviews (CATI), participant collaboration in at-home tasks (i.e., actigraphy), and a 2nd in-home visit. REGARDS oversampled Black individuals and residents of the southeastern United States known as the Stroke Belt and 17% reside in rural areas. REGARDS currently follows ~11,000 surviving participants. Comprehensive available data include adjudicated health events, social determinants of health (SDOH), cognition, biomarkers and genomics. Participants currently have mean age 76.9 (range 57-105), are 37% Black, have high cardiovascular risk, and 54% reside in the southeast — all factors associated with COVID-19 risk

and adverse outcomes. Participants are geocoded, and linked to administrative data such as EPA and Medicare. Biorepositories were assembled in 2003-2007 and 2013-2016.

Severe Asthma Research Program (SARP): SARP has been investigating the clinical, physiologic and molecular phenotypes of asthma since 2000. It is currently following ~400 deeply phenotyped asthma patients (60% severe), most with sputum samples, bronchoscopies, lung CTs, allergy status, spirometry and biobanking.

Subpopulations and Intermediate Outcome Measures in COPD Study (SPIROMICS): SPIROMICS is a multi-center, observational, longitudinal case-control study designed to guide future development of therapies for COPD by 1) providing robust criteria for sub-classifying COPD participants into groups most likely to benefit from a given therapy during a clinical trial, thereby improving the chances of successful outcome; and 2) identifying biomarkers and phenotypes that can be used as intermediate outcomes to reliably predict clinical benefit during therapeutic trials. The baseline exam included morphometric measures, spirometry, six-minute walk, an inspiratory and expiratory chest CT, and a set of standardized questionnaires. Biospecimens, including plasma, serum, DNA, urine and induced sputum, have been collected and stored. SPIROMICS has recruited 2,983 COPD cases and controls, 40-80 years old with 20+ pack-years of smoking at 12 US sites in 2010–2015.SPIROMICS has 5 follow-up exams, that include spirometry, lung CT scans, sputum induction and, in a subset, bronchoscopies; its current exam is ongoing.

Strong Heart Study (STRONG): STRONG was designed to respond to the recommendations from the Subcommittee on Cardiovascular and Cerebrovascular Disease of the Secretary of Health and Human Service's Task Force on Black and Minority Health that concluded that information on cardiovascular disease (CVD) in American Indians was inadequate. In its initial stages, the STRONG included three components. The first was a survey to determine cardiovascular disease mortality rates from 1984 to 1994 among tribal members aged 35-74 years of age residing in the 3 study areas (the community mortality study). The second was the clinical examination of 4,500 eligible tribal members. The third component is the morbidity and mortality (M&M) surveillance of these 4,500 participants. STRONG has completed three clinical examinations of the original Cohort in Phase I 1989-1991; Phase II: 1993-1995; 1998-1999, respectively. In Phases III-V, STRONG expanded to include genetic epidemiologic studies and family-based genetics studies due to the importance of genetics in the occurrence of CVD. Phase VI was a surveillance of the original STRONG cohort and of the STRONG family study participants to better understand CVD, cancer, liver disease, and inflammation in American Indians. Phase VII is currently underway with continued surveillance beginning February 2019 for a seven-year duration. The STRONG Phase VII exam serves as a platform for in-depth ancillary studies that are funded outside of the STRONG contracts.

Supplementary Table 1. Baseline characteristics of C4R participants not included in the analysis due to missing serosurvey.

Characteristic	Missing dried blood spot data (n=26584)
Age, no. (%)	
Less than 50 years	1759 (6.7%)
50-64 years	7441 (28.3%)
65-79 years	11881 (45.1%)
80 years and greater	5253 (19.9%)
Sex, no. (%)	
Female	15795 (59.5%)
Male	10772 (40.5%)
Self-reported race or ethnicity, no. (%)	
Non-Hispanic White	14134 (53.2%)
American Indian and Alaskan Native	772 (2.9%)
Asian	748 (2.8%)
African American/Black	5901 (22.2%)
Hispanic	5010 (18.9%)
Education attainment, no. (%)	
Less than high school	2645 (9.6%)
High school	5592 (21.7%)
College	5101 (19.8%)
Beyond college	12620 (49%)
Smoking status, no. (%)	
Never	13138 (49.9%)
Former	10040 (38.1%)
Current	3169 (12%)
Body mass index, no. (%)	
<25 kg/m^2	5969 (23.5%)
25-29.9 kg/m^2	9573 (37.6%)
30-35 kg/m^2	5858 (23%)
>35 kg/m^2	4036 (15.9%)
Hypertension, no. (%)	13541 (52%)
Diabetes, no. (%)	5105 (19.7%)
Cardiovascular disease, no. (%)	1324 (7.7%)
Chronic obstructive pulmonary disease, no. (%)	1610 (9.6%)
Asthma, no. (%)	2168 (12.7%)
Chronic kidney disease, no. (%)	638 (2.6%)
Vaccine type, no. (%)	
BNT162b2 mRNA (Pfizer-BioNTech)	13237 (50.5%)
mRNA-1273 (Moderna)	11431 (43.6%)
Other*	1549 (5.9%)
Study cohort, no. (%)	
ARIC	3092 (11.6%)
CARDIA	1494 (5.6%)
COPDGene	2109 (7.9%)
FHS	2762 (10.4%)
HCHS	4176 (15.7%)
JHS	1277 (4.8%)
MASALA	436 (1.6%)

MESA	1667 (6.3%)
NOMAS	813 (3.1%)
PrePF	432 (1.6%)
REGARDS	6937 (26.1%)
SARP	166 (0.6%)
SHS	748 (2.8%)
SPIROMICS	475 (1.8%)

Abbreviations: ARIC=Atherosclerosis Risk in Communities Study; CARDIA=Coronary Artery Risk Development in Young Adults, COPDGene=Genetic Epidemiology of Chronic Obstructive Pulmonary Disease, FHS=Framingham Heart Study; HCHS=Hispanic Community Health Study; JHS=Jackson Heart Study; MASALA=Mediators of Atherosclerosis in South Asians Living in America; MESA=Multi-Ethnic Study of Atherosclerosis; MFI=median fluorescence intensity; NOMAS=Northern Manhattan Study; PrePF=Preclinical Pulmonary Fibrosis; REGARDS=Reasons for Geographic and Racial Differences in Stroke; SHS=Strong Heart Study

Chronic kidney disease defined as estimated glomerular filtration rate below 45 $mL/min/1.73m^2$

*Received a vaccine other than BNT162b2 mRNA or mRNA-1273 or did not know the type of vaccine received.

Supplementary Table 2. Proportion of C4R participants with anti-S1 reactivity, overall and by selected characteristics.

	Time between vaccine dose and serosurvey				
**Group**	0-30 days (n=107)	31-60 days (n=931)	61-120 days (n=1934)	121-180 days (n=2238)	181-270 days (n=1035)
Overall	100 (93.5%)	900 (96.7%)	1908 (98.7%)	2185 (97.6%)	993 (95.9%)
Vaccine type					
BNT162b2 (Pfizer)	69/75 (92%)	481/500 (96.2%)	981/1000 (98.1%)	1076/1110 (96.9%)	518/552 (93.8%)
mRNA-1273 (Moderna)	31/32 (96.9%)	419/431 (97.2%)	927/934 (99.3%)	1109/1128 (98.3%)	475/483 (98.3%)
Age group					
Less than 65 years	33/35 (94.3%)	168/171 (98.2%)	453/457 (99.1%)	552/557 (99.1%)	210/221 (95%)
65-79 years	35/37 (94.6%)	347/359 (96.7%)	859/870 (98.7%)	1259/1289 (97.7%)	601/620 (96.9%)
80 years and greater	32/35 (91.4%)	385/401 (96%)	593/604 (98.2%)	364/382 (95.3%)	179/191 (93.7%)

Abbreviations: Results are presented by time since serosurvey and the % reported are calculated as the number reactive in each respective group of interest (i.e., overall, vaccine type, or age group) in the numerator and the total number in each respective group of interest in the denominator.

Supplementary Table 3. Associations of risk factors with changes in anti-S1 IgG levels 60 days after vaccination

**Risk factors**	**Mean % change in anti-S1 IgG level rate of change per 30 days (95% CI)**
Male (ref. female)	-1.4 (-4.5 to 1.9)
Age	
65-79 years (ref. less than 65 years)	2.5 (-1.8 to 7.1)
80 years and greater (ref. less than 65 years)	-1.5 (-6.2 to 3.5)
Race/ethnicity	
American Indian Alaskan Native (ref. non-Hispanic White)	7.1 (-5.8 to 22.0)
Asian (ref. non-Hispanic White)	-1.7 (-12.0 to 9.9)
African American or Black (ref. non-Hispanic White)	2.3 (-2.0 to 6.7)
Hispanic (ref. non-Hispanic White)	-8.7 (-24.0 to 9.7)
Smoking	
Former (ref. never)	-2.5 (-5.7 to 0.9)
Current (ref. never)	-6.8 (-12.1 to -1.0)
Education attainment	
Less than high school (ref. college or beyond)	6.7 (-1.8 to 16.0)
High school (ref. college or beyond)	0.9 (-3.1 to 5.1)
Some college (ref. college or beyond)	3.0 (-1.5 to 7.8)
Body mass index	
25-29.9 kg/m^2 (ref. less than 25 kg/m^2)	1.0 (-3.2 to 5.3)
30-34.9 kg/m^2 (ref. less than 25 kg/m^2)	6.2 (1.3 to 11.4)
>35 kg/m^2 (ref. less than 25 kg/m^2)	2.0 (-3.5 to 7.9)
Diabetes (ref. no diabetes)	3.3 (-0.8 to 7.6)
Hypertension (ref. no hypertension)	0.3 (-3.0 to 3.6)
Cardiovascular disease (ref. no cardiovascular disease)	2.3 (-4.6 to 9.7)
Chronic kidney disease (ref. no chronic kidney disease)	7.4 (-5.2 to 21.4)
Asthma (ref. no asthma)	-4.1 (-9.2 to 1.2)
COPD (ref. no COPD)	-1.9 (-8.1 to 4.8)
Log-transformed anti-N MFI (per 1-unit increment)	5.2 (3.8 to 6.7)
COVID-19 infection prior to serosurvey collection	
Infection prior to vaccine-not hospitalized (ref. no infection)	5.8 (-0.2 to 12.1)
Infection prior to vaccine- hospitalized (ref. no infection)	9.2 (-2.8 to 22.6)
Infection after vaccine (ref. no infection)	12.6 (3.1 to 22.9)
Vaccine type	
BNT162b2 mRNA- Pfizer-BioNTech (ref. mRNA-1273-Moderna)	-3.7 (-6.8 to -0.6)

Abbreviations: CI=confidence interval; COPD=chronic obstructive pulmonary disease; MFI=median fluorescence intensity
Linear spline model adjusted for age, sex, race/ethnicity, smoking history, education attainment, body mass index, diabetes, cardiovascular disease, asthma, chronic obstructive pulmonary disease, anti-N, COVID-19 infection history, dried blood spot batch, vaccine type, and vaccine dose.
Chronic kidney disease defined as estimated glomerular filtration rate below 45 $mL/min/1.73m^2$

Supplementary Table 4. Complete case analysis.

Clinical risk factor	Estimated mean percent difference in anti-S1 Iglevel (95% confidence interval)	P-value
Age		
Less than 65 years	0.0 (ref)	
65-79 years	-17.6 (-27.2 to -6.8)	0.002
80 years and greater	-37.3 (-45.1 to -28.4)	7.00e10^{-12}
Sex		
Female	0.0 (ref)	
Male	-18.9 (-25.9 to -11.3)	4.78e10^{-6}
Race/ethnicity		
Non-Hispanic white	0.0 (ref)	
American Indian or Alaskan Native	-1.3 (-30.4 to 39.9)	0.94
Asian	-23.8 (-66.1 to 71.3)	0.51
African American or Black	2.5 (-11.2 to 18.3)	0.74
Hispanic	-14.9 (52.0 to 51.1)	0.58
Education attainment		
College or beyond	0.0 (ref)	
Less than high school	-4.0 (-23.4 to 20.2)	0.72
High school	-1.7 (-11.8 to 9.6)	0.76
Some college	-5.7 (-17.1 to 7.1)	0.37
Smoking history		
Never	0.0 (ref)	
Former	-2.4 (-10.8 to 6.9)	0.61
Current	-5.2 (-20.5 to 13.2)	0.56
Body mass index		
<25 kg/m^2	0.0 (ref)	
25-29.9 kg/m^2	10.3 (-0.8 to 22.8)	0.07
30-34.9 kg/m^2	1.1 (-11.0 to 14.8)	0.87
>35 kg/m^2	-6.6 (-20.1 to 9.2)	0.39
Diabetes		
No	0.0 (ref)	
Yes	-15.0 (-24.3 to -4.6)	0.006
Hypertension		
No	0.0 (ref)	
Yes	-0.5 (-9.1 to 8.9)	0.91
Cardiovascular disease		
No	0.0 (ref)	
Yes	-9.0 (-21.7 to 6.0)	0.23
Chronic kidney disease		
No	0.0 (ref)	
Yes	-14.6 (-32.8 to 8.5)	0.20
Asthma		
No	0.0 (ref)	
Yes	-4.8 (-17.1 to 9.2)	0.48
Chronic obstructive pulmonary disease		
No	0.0 (ref)	
Yes	-18.9 (-31.5 to -4.1)	0.01
Log-transformed anti-N MFI (per 1-unit increment)	30.2 (24.0 to 36.8)	2.16e10^{-25}
COVID-19 infection prior to serosurvey collection		
No infection	0.0 (ref)	
Infection prior to vaccine-not hospitalized	45.2 (18.6 to 77.7)	2.39e10^{-4}
Infection prior to vaccine- hospitalized	100.8 (47.1 to 174.0)	1.18e10^{-5}
Infection after vaccine	-3.5 (-26.7 to 26.9)	0.80
COVID-19 vaccine type		
BNT162b2 mRNA (Pfizer-BioNTech)	0.0 (ref)	
mRNA-1273 (Moderna)	75.2 (61.1 to 90.6)	8.43 e10^{-38}
Time between vaccine and serosurvey collection (per 30 days)	-12.2 (-16.0 to -8.2)	9.19e10^{-9}

Abbreviations: MFI=median fluorescence intensity

Chronic kidney disease defined as estimated glomerular filtration rate below 45 mL/min/1.73m
Results arise from a linear regression model including all variables present in this table.
Statistical significance was assessed using two-sided tests. P-values are reported without adjustment for multiple comparisons.

Supplementary Table 5. Multivariable adjusted correlates of anti-S1 IgG levels after COVID-19 vaccination after excluding 284 non-definite cases of prior SARS-CoV-2.

Clinical risk factor	Estimated mean percent difference in anti-S1 IgG level (95% confidence interval)	P-value
Age		
Less than 65 years	0.0 (ref)	
65-79 years	-20.8 (-26.4 to -14.8)	$5.81e10^{-10}$
80 years and greater	-40.7 (-45.6 to -35.3)	$3.00e10^{-31}$
Sex		
Female	0.0 (ref)	
Male	-23.0 (-27.2 to -18.5)	$1.03e10^{-19}$
Race/ethnicity		
Non-Hispanic white	0.0 (ref)	
American Indian or Alaskan Native	-26.6 (-41.2 to -8.4)	0.006
Asian	34.7 (15.3 to 57.6)	$1.89e10^{-4}$
African American or Black	2.4 (-5.1 to 10.5)	0.54
Hispanic	13.4 (-15.1 to 51.6)	0.40
Education attainment		
College or beyond	0.0 (ref)	
Less than high school	-3.2 (-16.3 to 12.0)	0.66
High school	-1.7 (-8.3 to 5.5)	0.64
Some college	-2.4 (-9.2 to 4.9)	0.51
Smoking history		
Never	0.0 (ref)	
Former	-9.5 (-14.8 to -4.0)	$9.00e10^{-4}$
Current	-20.1 (-27.7 to -11.5)	$1.56e10^{-5}$
Body mass index		
<25 kg/m^2	0.0 (ref)	
25-29.9 kg/m^2	7.6 (0.2 to 15.4)	0.04
30-34.9 kg/m^2	3.8 (-4.3 to 12.5)	0.37
>35 kg/m^2	-10.1 (-18.5 to -1.0)	0.03
Diabetes		
No	0.0 (ref)	
Yes	-13.2 (-19.3 to -6.7)	$1.41e10^{-4}$
Hypertension		
No	0.0 (ref)	
Yes	-4.5 (-9.9 to 1.2)	0.12
Cardiovascular disease		
No	0.0 (ref)	
Yes	-5.6 (-14.7 to 4.4)	0.26
Chronic kidney disease		
No	0.0 (ref)	
Yes	-10.5 (-26.2 to 8.7)	0.26
Asthma		
No	0.0 (ref)	
Yes	-6.9 (-15.6 to 2.8)	0.16
Chronic obstructive pulmonary disease		
No	0.0 (ref)	
Yes	-18.3 (-27.3 to -8.2)	0.001
Log-transformed anti-N MFI (per 1-unit increment)	36.8 (32.4 to 41.2)	$1.02e10^{-68}$
COVID-19 infection prior to serosurvey collection		
No infection	0.0 (ref)	
Infection prior to vaccine-not hospitalized	65.4 (49.5 to 82.9)	$2.03e10^{-20}$
Infection prior to vaccine- hospitalized	84.8 (54.2 to 121.2)	$9.01e10^{-10}$
Infection after vaccine	8.1 (-17.0 to 40.9)	0.56
COVID-19 vaccine type		
BNT162b2 mRNA (Pfizer-BioNTech)	0.0 (ref)	
mRNA-1273 (Moderna)	91.7 (81.7 to 102.2)	$3.48e10^{-120}$

Time between vaccine and serosurvey collection (per 30 days)	-17.1 (-19.2 to -15.0)	$4.22e10^{-55}$

Abbreviations: MFI=median fluorescence intensity
Chronic kidney disease defined as estimated glomerular filtration rate below 45 mL/min/1.73m
"Definite infection" criteria used to categorize participants to having a history of COVID-19 infection prior to dried blood spot collection: Proxy/self-report of positive test for SARS-CoV-2; anti-nucleocapsid protein IgG on C4R serology regardless of COVID vaccination; anti-spike protein IgG on C4R serology without COVID vaccination.
Results arise from a linear regression model including all variables present in this table.
Statistical significance was assessed using two-sided tests. P-values are reported without adjustment for multiple comparisons.

Supplementary Table 6. Correlates of anti-S1 IgG levels after COVID-19 vaccination in multivariable adjusted analyses including cohort adjustment. Results from n=6,245 C4R participants with serology results from February 2021 through August 2022.

Clinical risk factor	Estimated mean percent difference in anti-S1 IgG level (95% confidence interval)	P-value
Age		
Less than65 years	0.0 (ref)	
65-79 years	-16.8 (-23.3 to -9.9)	$7.50e10^{-6}$
80 years and greater	-37.6 (-43.5 to -30.9)	$5.09e10^{-20}$
Sex		
Female	0.0 (ref)	
Male	-20.8 (-25.0 to -16.3)	$1.35e10^{-16}$
Race/ethnicity		
Non-Hispanic white	0.0 (ref)	
American Indian or Alaskan Native	-9.2 (-56.4 to 89.5)	0.80
Asian	-12.8 (-35.9 to 18.8)	0.39
African American or Black	8.7 (0.6 to 17.4)	0.04
Hispanic	4.3 (22.6 to 40.4)	0.78
Education attainment		
College or beyond	0.0 (ref)	
Less than high school	2.5 (-11.0 to 18.2)	0.73
High school	2.3 (-4.6 to 9.7)	0.52
Some college	0.2 (-6.7 to 7.6)	0.96
Smoking history		
Never	0.0 (ref)	
Former	-8.6 (-13.8 to -3.1)	0.003
Current	-17.1 (-25.0 to -8.3)	$2.66e10^{-4}$
Body mass index		
<25 kg/m^2	0.0 (ref)	
25-29.9 kg/m^2	6.1 (-1.0 to 13.7)	0.09
30-34.9 kg/m^2	2.4 (-5.4 to 10.8)	0.56
>35 kg/m^2	-11.8 (-19.8 to -3.0)	0.01
Diabetes		
No	0.0 (ref)	
Yes	-12.7 (-18.7 to -6.3)	$1.84e10^{-4}$
Hypertension		
No	0.0 (ref)	
Yes	-2.8 (-8.1 to 2.9)	0.34
Cardiovascular disease		
No	0.0 (ref)	
Yes	-7.3 (-16.1 to 2.4)	0.14
Chronic kidney disease		
No	0.0 (ref)	
Yes	-8.7 (-24.9 to 10.8)	0.36
Asthma		
No	0.0 (ref)	
Yes	-8.0 (-16.8 to 1.8)	0.11
Chronic obstructive pulmonary disease		
No	0.0 (ref)	
Yes	-13.3 (-23.0 to -2.6)	0.02
Log-transformed anti-N MFI (per 1-unit increment)	35.8 (31.5 to 40.2)	$3.59e10^{-52}$
COVID-19 infection prior to serosurvey collection		
No infection	0.0 (ref)	
Infection prior to vaccine-not hospitalized	75.9 (58.9 to 94.8)	$1.08e10^{-22}$
Infection prior to vaccine- hospitalized	100.4 (65.0 to 143.5)	$6.18e10^{-10}$
Infection after vaccine	6.9 (-11.4 to 29.0)	0.49
COVID-19 vaccine type		
BNT162b2 mRNA (Pfizer-BioNTech)	0.0 (ref)	
mRNA-1273 (Moderna)	87.2 (77.5 to 97.2)	$1.48e10^{-116}$

Time between vaccine and serosurvey collection (per 30 days)	-16.5 (-18.5 to -14.4)	$1.46e10^{-44}$
Study cohort		
ARIC	0.0 (ref)	
CARDIA	-25.8 (-41.1 to -6.7)	0.01
COPDGene	-29.9 (-40.1 to -17.8)	$1.39e10^{-5}$
FHS	12.0 (1.4 to 23.6)	0.03
JHS	13.0 (-22.0 to 63.6)	0.52
MASALA	69.7 (17.9 to 144.2)	0.004
MESA	-15.2 (-30.2 to 2.9)	0.10
PrePF	-9.5 (-30.9 to 18.3)	0.46
REGARDS	-15.2 (-23.7 to -5.8)	0.002
SARP	-25.8 (-45.8 to 1.6)	0.06
SHS	-34.8 (-69.9 to 41.3)	0.28
SPIROMICS	-6.6 (-26.4 to 18.6)	0.58

Abbreviations: MFI=median fluorescence intensity
Chronic kidney disease defined as estimated glomerular filtration rate below 45 mL/min/1.73m
Results arise from a linear regression model including all variables present in this table.
Statistical significance was assessed using two-sided tests. P-values are reported without adjustment for multiple comparisons.

Supplementary Table 7. Classification of definite versus probable SARS-CoV-2 infection in C4R.

Definite Infection	Probable Infection
• Adjudicated definite COVID-19 infection<br>• Proxy/self-report of positive test for SARS-CoV-2<br>• Anti-nucleocapsid protein IgG on C4R serology, regardless of COVID vaccination<br>• Anti-spike protein IgG on C4R serology without COVID vaccination	• Adjudicated probable COVID-19 infection<br>• Proxy/self-report of self- or healthcare provider-diagnosed COVID-19 without confirmatory testing

Supplementary Table 8. Number of participants with missing covariate data.

Characteristic	No. Participants, %
Total number of participants	6245, 100%
Age	16, 0.3%
Sex	1, 0.02%
Self-reported race or ethnicity	4, 0.06%
Education attainment	174, 2.8%
Smoking status	36, 0.6%
Body mass index	213, 3.4%
Hypertension	31, 0.5%
Diabetes	40, 0.6%
Cardiovascular disease	2531, 40.5%
Chronic obstructive pulmonary disease	2349, 37.6%
Asthma	2445, 39.2%
Chronic kidney disease	541, 8.7%
COVID-19 infection prior to serosurvey	2250, 36.0%
Vaccine type	0
Time between first vaccination dose and serosurvey	0

Chronic kidney disease defined as estimated glomerular filtration rate below 45 mL/min/1.73m^2

Supplementary Table 9. Baseline characteristics of non-imputed data

		Anti-S1 Quartiles			
**Characteristic**	**Overall**	**Quartile 1**	**Quartile 2**	**Quartile 3**	**Quartile 4**
No. of participants	6245	1562	1561	1561	1561
Anti-S1 antibody MFI (log-transformed)	8.4 (1.3)	6.6 (1.0)	8.1 (0.3)	9.0 (0.3)	10.0 (0.3)
Natural log transformed-anti-N antibody MFI (SE)	4.7 (1.1)	4.3 (0.8)	4.5 (0.9)	4.7 (1.0)	5.3 (1.5)
Age					
Less than 65 years	1441 (23.1%)	233 (15%)	373 (24%)	428 (27.5%)	407 (26.1%)
65-79 years	3175 (51%)	866 (55.6%)	844 (54.2%)	745 (47.8%)	3175 (51%)
80 years and greater	1613 (25.9%)	458 (29.4%)	340 (21.8%)	384 (24.7%)	431 (27.7%)
Female sex	3642 (58.3%)	784 (50.2%)	906 (58%)	963 (61.7%)	989 (63.4%)
Self-reported race or ethnicity					
Non-Hispanic White	4769 (76.4%)	1218 (78%)	1171 (75.1%)	1196 (76.6%)	1184 (75.9%)
African American or Black	1098 (17.6%)	271 (17.4%)	302 (19.4%)	258 (16.5%)	267 (17.1%)
Hispanic	56 (0.9%)	14 (0.9%)	14 (0.9%)	15 (1%)	13 (0.8%)
Asian	193 (3.1%)	28 (1.8%)	51 (3.3%)	66 (4.2%)	48 (3.1%)
American Indian and Alaskan Native	125 (2%)	30 (1.9%)	22 (1.4%)	26 (1.7%)	47 (3%)
Education attainment					
Less than high school	242 (4%)	52 (3.4%)	59 (3.9%)	56 (3.7%)	75 (5%)
High school	1334 (22%)	330 (21.7%)	299 (19.6%)	324 (21.4%)	381 (25.3%)
Some college	1193 (19.7%)	323 (21.3%)	355 (23.2%)	293 (19.3%)	222 (14.7%)
College or beyond	3302 (54.4%)	814 (53.6%)	814 (53.3%)	844 (55.6%)	830 (55%)
Study cohort					
ARIC	1705 (27.3%)	253 (16.2%)	286 (18.3%)	491 (31.5%)	675 (43.2%)
CARDIA	144 (2.3%)	34 (2.2%)	49 (3.1%)	36 (2.3%)	25 (1.6%)
COPDGene	444 (7.1%)	144 (9.2%)	132 (8.5%)	93 (6%)	75 (4.8%)
FHS	1138 (18.2%)	127 (8.1%)	258 (16.5%)	326 (20.9%)	427 (27.4%)
JHS	34 (0.5%)	7 (0.4%)	6 (0.4%)	14 (0.9%)	7 (0.4%)
MASALA	145 (2.3%)	12 (0.8%)	40 (2.6%)	57 (3.7%)	36 (2.3%)
MESA	215 (3.4%)	77 (4.9%)	65 (4.2%)	52 (3.3%)	21 (1.3%)
PrePF	72 (1.2%)	24 (1.5%)	18 (1.2%)	16 (1%)	14 (0.9%)
REGARDS	2087 (33.4%)	811 (51.9%)	649 (41.6%)	418 (26.8%)	209 (13.4%)
SARP	48 (0.8%)	14 (0.9%)	12 (0.8%)	9 (0.6%)	13 (0.8%)
SHS	117 (1.9%)	28 (1.8%)	20 (1.3%)	24 (1.5%)	45 (2.9%)
SPIROMICS	96 (1.5%)	31 (2%)	26 (1.7%)	25 (1.6%)	14 (0.9%)
Smoking status					
Never	2819 (45.4%)	631 (40.5%)	707 (45.4%)	758 (49%)	723 (46.7%)
Former	2815 (45.3%)	762 (48.9%)	690 (44.3%)	663 (42.8%)	700 (45.2%)
Current	575 (9.3%)	164 (10.5%)	160 (10.3%)	127 (8.2%)	124 (8%)
Body mass index, kg/m^2					

<25 kg/m^2	1528 (25.3%)	393 (25.7%)	375 (25%)	362 (24.3%)	398 (26.3%)
25-29.9 kg/m^2	2353 (39%)	571 (37.3%)	583 (38.8%)	604 (40.6%)	595 (39.4%)
30-35 kg/m^2	1340 (22.2%)	334 (21.8%)	352 (23.4%)	320 (21.5%)	334 (22.1%)
>35 kg/m^2	811 (13.4%)	231 (15.1%)	193 (12.8%)	202 (13.6%)	185 (12.2%)
Hypertension	3418 (55%)	966 (62.2%)	839 (54%)	801 (51.5%)	812 (52.3%)
Diabetes	1198 (19.3%)	382 (24.6%)	287 (18.5%)	255 (16.4%)	274 (17.7%)
Cardiovascular disease	331 (8.9%)	73 (11%)	80 (10%)	81 (8%)	97 (7.9%)
Chronic obstructive pulmonary disease	399 (10.2%)	108 (15.3%)	96 (11.4%)	98 (9.3%)	97 (7.5%)
Asthma	449 (11.8%)	101 (14.9%)	98 (12%)	98 (9.5%)	152 (11.9%)
Chronic kidney disease	135 (2.4%)	34 (2.4%)	34 (2.4%)	36 (2.5%)	31 (2.2%)
COVID-19 infection prior to serosurvey					
No infection	3281 (82.1%)	650 (91.4%)	751 (89.4%)	917 (86%)	963 (69.9%)
Infection prior to vaccine-not hospitalized	403 (10.1%)	34 (4.8%)	55 (6.5%)	92 (8.6%)	222 (16.1%)
Infection prior to vaccine-hospitalized	96 (2.4%)	9 (1.3%)	3 (0.4%)	18 (1.7%)	66 (4.8%)
Infection after vaccine-not hospitalized	215 (5.4%)	18 (2.5%)	31 (3.7%)	39 (3.7%)	127 (9.2%)
Vaccine type					
BNT162b2 mRNA (Pfizer-BioNTech)	3237 (51.8%)	1080 (69.1%)	823 (52.7%)	702 (45%)	632 (40.5%)
mRNA-1273 (Moderna)	3008 (48.2%)	482 (30.9%)	738 (47.3%)	859 (55%)	929 (59.5%)
Time between vaccination and serosurvey, months	4.0 (1.7)	5.0 (1.5)	4.6 (1.5)	3.8 (1.6)	2.8 (1.5)

Abbreviations: ARIC=Atherosclerosis Risk in Communities Study; CARDIA=Coronary Artery Risk Development in Young Adults, COPDGene=Genetic Epidemiology of Chronic Obstructive Pulmonary Disease, FHS=Framingham Heart Study; JHS=Jackson Heart Study; MASALA=Mediators of Atherosclerosis in South Asians Living in America; MESA=Multi-Ethnic Study of Atherosclerosis; MFI=median fluorescence intensity; PrePF=Preclinical Pulmonary Fibrosis; REGARDS=Reasons for Geographic and Racial Differences in Stroke; SHS=Strong Heart Study

Continuous variables presented as mean (standard deviation)
Categorical variables presented as number of participants (percentage)
Percentages rounded to nearest tenth decimal point
Chronic kidney disease defined as estimated glomerular filtration rate below 45 mL/min/1.73m^2

Supplementary Figure 1. Flow chart of participants included in the analysis.

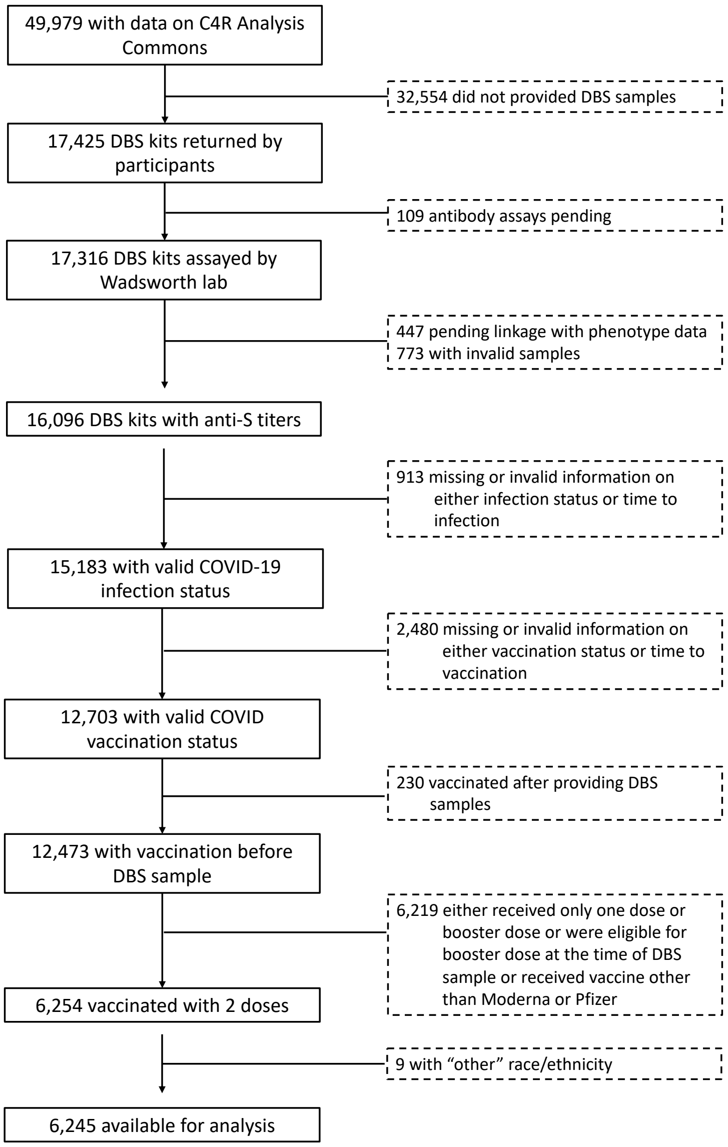

Supplementary Figure 2. Comparison of associations in cohort-stratified models. Abbreviations: MFI=median fluorescence intensity. Chronic kidney disease defined as estimated glomerular filtration rate below 45 mL/min/1.73m. Results arise from a linear regression model including all variables present in the figures. Statistical significance was assessed using two-sided tests.

2a

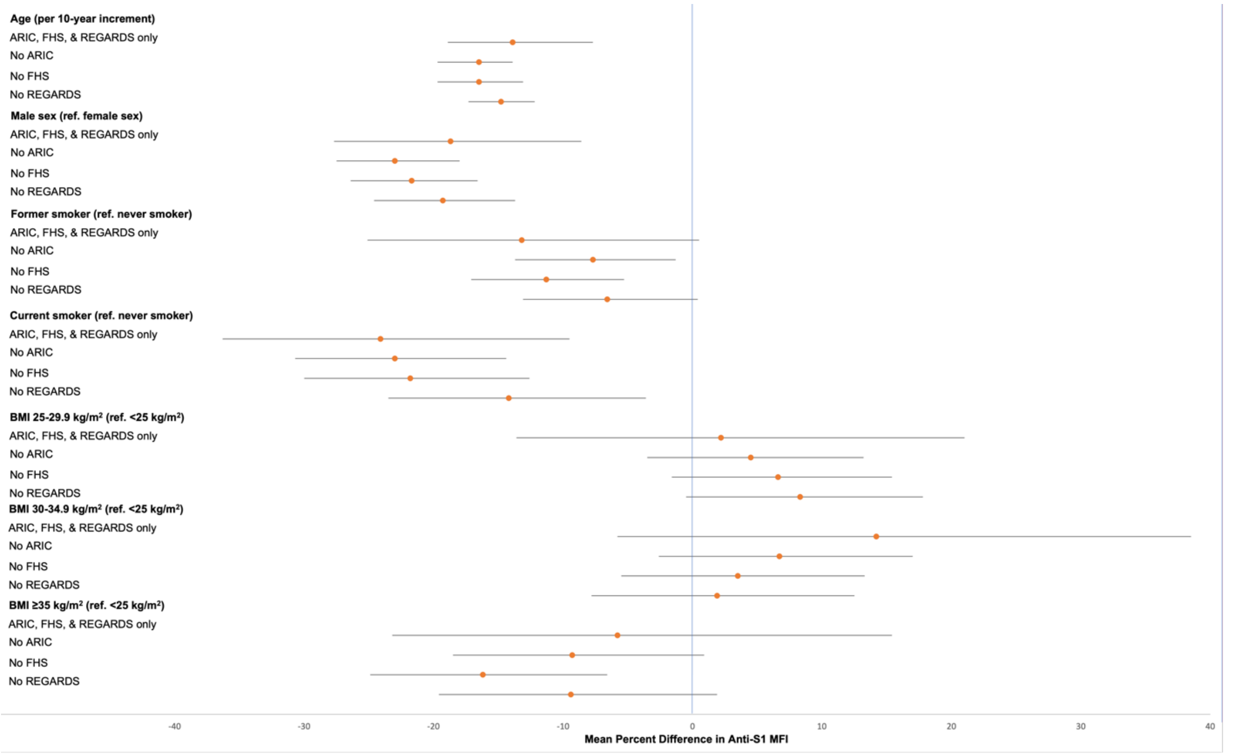

2b

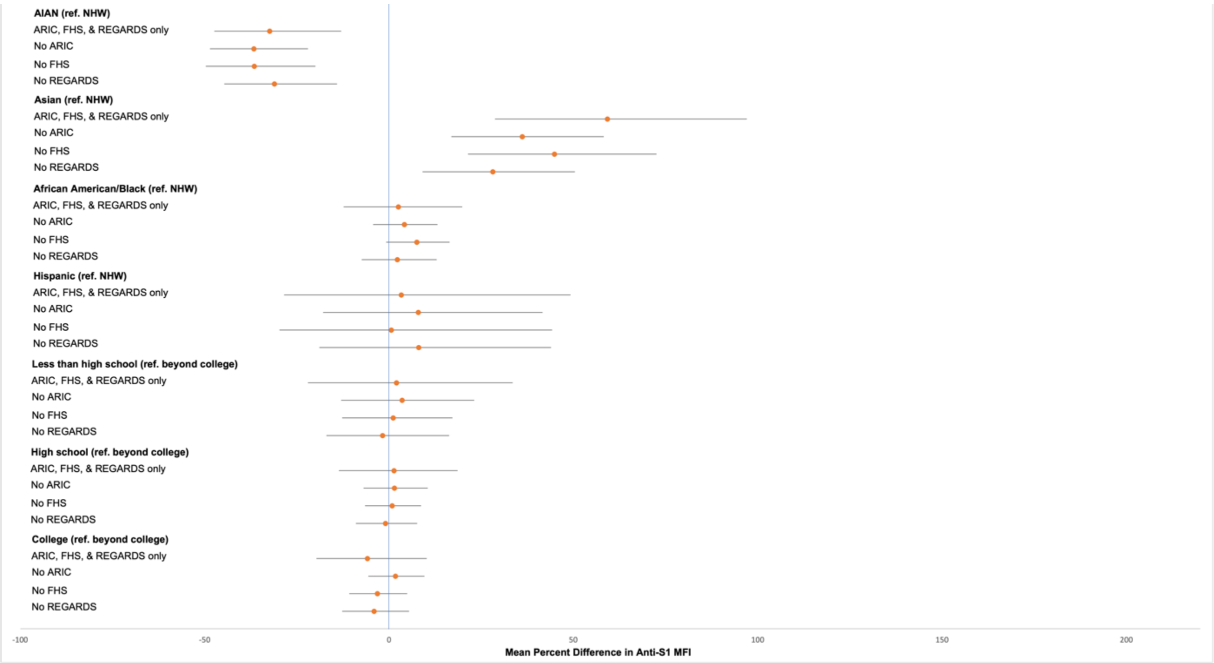

AIAN (ref. NHW)
ARIC, FHS, & REGARDS only
No ARIC
No FHS
No REGARDS
Asian (ref. NHW)
ARIC, FHS, & REGARDS only
No ARIC
No FHS
No REGARDS
African American/Black (ref. NHW)
ARIC, FHS, & REGARDS only
No ARIC
No FHS
No REGARDS
Hispanic (ref. NHW)
ARIC, FHS, & REGARDS only
No ARIC
No FHS
No REGARDS
Less than high school (ref. beyond college)
ARIC, FHS, & REGARDS only
No ARIC
No FHS
No REGARDS
High school (ref. beyond college)
ARIC, FHS, & REGARDS only
No ARIC
No FHS
No REGARDS
College (ref. beyond college)
ARIC, FHS, & REGARDS only
No ARIC
No FHS
No REGARDS
-100
-50
0
50
100
150
200
Mean Percent Difference in Anti-S1 MFI

2c

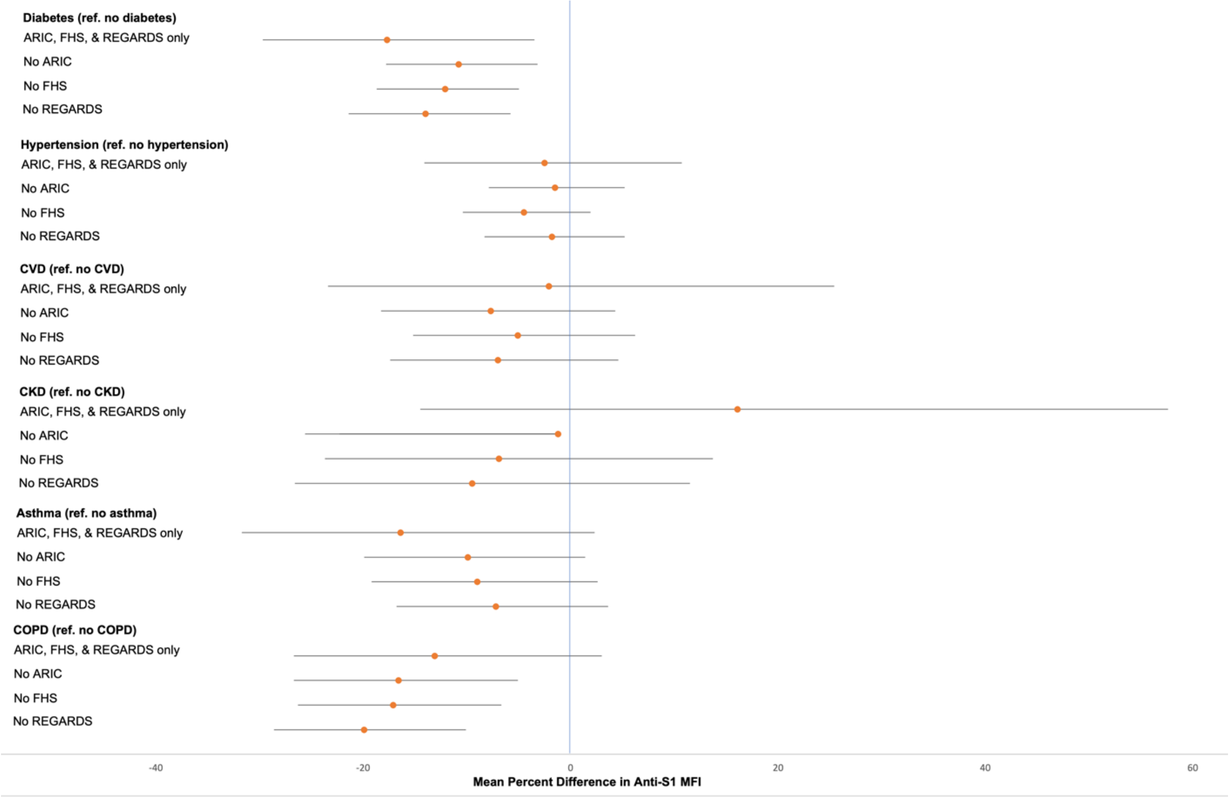

Diabetes (ref. no diabetes)
ARIC, FHS, & REGARDS only
No ARIC
No FHS
No REGARDS
Hypertension (ref. no hypertension)
ARIC, FHS, & REGARDS only
No ARIC
No FHS
No REGARDS
CVD (ref. no CVD)
ARIC, FHS, & REGARDS only
No ARIC
No FHS
No REGARDS
CKD (ref. no CKD)
ARIC, FHS, & REGARDS only
No ARIC
No FHS
No REGARDS
Asthma (ref. no asthma)
ARIC, FHS, & REGARDS only
No ARIC
No FHS
No REGARDS
COPD (ref. no COPD)
ARIC, FHS, & REGARDS only
No ARIC
No FHS
No REGARDS
-40
-20
0
20
40
60
Mean Percent Difference in Anti-S1 MFI

2d

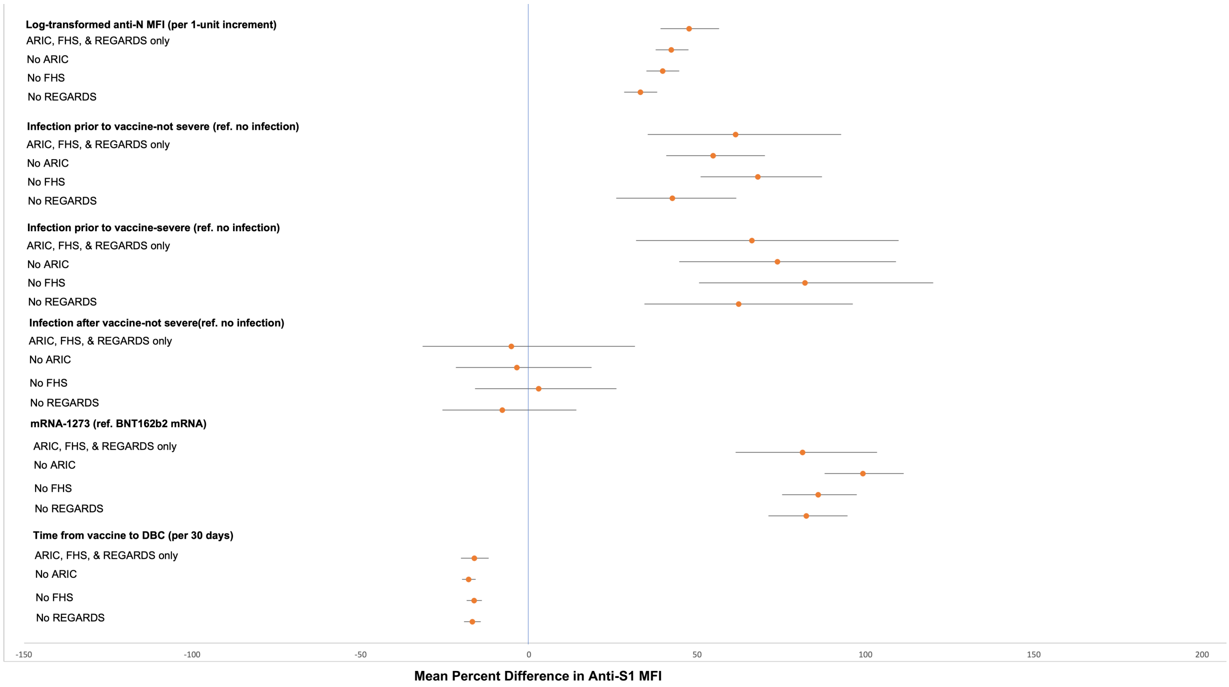

Log-transformed anti-N MFI (per 1-unit increment)
ARIC, FHS, & REGARDS only
No ARIC
No FHS
No REGARDS
Infection prior to vaccine-not severe (ref. no infection)
ARIC, FHS, & REGARDS only
No ARIC
No FHS
No REGARDS
Infection prior to vaccine-severe (ref. no infection)
ARIC, FHS, & REGARDS only
No ARIC
No FHS
No REGARDS
Infection after vaccine-not severe(ref. no infection)
ARIC, FHS, & REGARDS only
No ARIC
No FHS
No REGARDS
mRNA-1273 (ref. BNT162b2 mRNA)
ARIC, FHS, & REGARDS only
No ARIC
No FHS
No REGARDS
Time from vaccine to DBC (per 30 days)
ARIC, FHS, & REGARDS only
No ARIC
No FHS
No REGARDS
-150
-100
-50
0
50
100
150
200
Mean Percent Difference in Anti-S1 MFI
