# Supplementary material for: Demographic and Clinical Factors Associated With SARS-CoV-2 Spike 1 Antibody Response Among Vaccinated US Adults: the C4R Study
Source: Nat Commun. 2024 Feb 19;15:1492. doi: 10.1038/s41467-024-45468-9 (PMC10876680; doi:10.1038/s41467-024-45468-9)
Supplement: Supplementary file 3 — Reporting Summary [file 41467_2024_45468_MOESM3_ESM.pdf]

# nature portfolio

Corresponding author(s): John Kim

Last updated by author(s): Jan 19, 2024



# Reporting Summary

Nature Portfolio wishes to improve the reproducibility of the work that we publish. This form provides structure for consistency and transparency in reporting. For further information on Nature Portfolio policies, see our Editorial Policies and the Editorial Policy Checklist.

## Statistics

For all statistical analyses, confirm that the following items are present in the figure legend, table legend, main text, or Methods section.

| n/a | Confirmed | |
|---|---|---|
| ☐ | ☒ | The exact sample size (*n*) for each experimental group/condition, given as a discrete number and unit of measurement |
| ☐ | ☒ | A statement on whether measurements were taken from distinct samples or whether the same sample was measured repeatedly |
| ☐ | ☒ | The statistical test(s) used AND whether they are one- or two-sided<br>*Only common tests should be described solely by name; describe more complex techniques in the Methods section.* |
| ☐ | ☒ | A description of all covariates tested |
| ☐ | ☒ | A description of any assumptions or corrections, such as tests of normality and adjustment for multiple comparisons |
| ☐ | ☒ | A full description of the statistical parameters including central tendency (e.g. means) or other basic estimates (e.g. regression coefficient) AND variation (e.g. standard deviation) or associated estimates of uncertainty (e.g. confidence intervals) |
| ☐ | ☒ | For null hypothesis testing, the test statistic (e.g. *F*, *t*, *r*) with confidence intervals, effect sizes, degrees of freedom and *P* value noted<br>*Give P values as exact values whenever suitable.* |
| ☒ | ☐ | For Bayesian analysis, information on the choice of priors and Markov chain Monte Carlo settings |
| ☒ | ☐ | For hierarchical and complex designs, identification of the appropriate level for tests and full reporting of outcomes |
| ☒ | ☐ | Estimates of effect sizes (e.g. Cohen's *d*, Pearson's *r*), indicating how they were calculated |

*Our web collection on statistics for biologists contains articles on many of the points above.*

## Software and code

Policy information about availability of computer code

| | |
|---|---|
| Data collection | Code for data cleaning and analysis is available, upon request and with appropriate consortium and cohort permissions, on the C4R Analysis Commons. Data harmonization for C4R performed using SAS Studio (SAS Data Science) in Seven Bridges Platform. |
| Data analysis | Analysis performed using R Studio (SB Bioinformatics-R 4.1). R packages used for analysis include: "tidyverse", "ggplot2", "arsenal", "mice", "mgcv", and "lspline". |

For manuscripts utilizing custom algorithms or software that are central to the research but not yet described in published literature, software must be made available to editors and reviewers. We strongly encourage code deposition in a community repository (e.g. GitHub). See the Nature Portfolio guidelines for submitting code & software for further information.

## Data

Policy information about availability of data

All manuscripts must include a data availability statement. This statement should provide the following information, where applicable:

- Accession codes, unique identifiers, or web links for publicly available datasets
- A description of any restrictions on data availability
- For clinical datasets or third party data, please ensure that the statement adheres to our policy

All data supporting the findings described in this manuscript are available in the article and in the Supplementary Information. Data is available, upon request and with appropriate consortium and cohort permissions, on the C4R Analysis Commons. Preliminary responses to data requests will be made within 4 weeks of receipt.

C4R Analysis Commons, hosted on BioData Catalyst powered by Seven Bridges (https://accounts.sb.biodatacatalyst.nhlbi.nih.gov/). Further information also available at the C4R website: https://c4r-nih.org.

## Research involving human participants, their data, or biological material

Policy information about studies with human participants or human data. See also policy information about sex, gender (identity/presentation), and sexual orientation and race, ethnicity and racism.

| | |
|---|---|
| Reporting on sex and gender | All sexes were considered in this observational cohort study and the sex of participants was determined based on self-report as documented within the respective cohorts. |
| Reporting on race, ethnicity, or other socially relevant groupings | All races and ethnicities were considered in this observational cohort study and the race and ethnicity of participants was determined based on self-report as documented within the respective cohorts. |
| Population characteristics | Human adults in the United States with a mean age of 73 years (range 21-100), 58% are female, and 76% are non-Hispanic White. |
| Recruitment | Cohort participants previously consented to in-person, telephone, and/or e-mail contact and for abstraction of medical records. Additional consent for ascertainment of COVID-19 data, including the serosurvey, is obtained according to cohort-specific procedures, including verbal, remote, and traditional written informed consent. |
| Ethics oversight | The administrative coordinating center is the NHLBI Collaborating Network of Networks for Evaluating COVID-19 and Therapeutic Strategies (CONNECTS) Program, which reviews C4R operational milestones biweekly. Central C4R functions are overseen by an observational studies monitoring board convened by CONNECTS. |

Note that full information on the approval of the study protocol must also be provided in the manuscript.

# Field-specific reporting

Please select the one below that is the best fit for your research. If you are not sure, read the appropriate sections before making your selection.

☒ Life sciences ☐ Behavioural & social sciences ☐ Ecological, evolutionary & environmental sciences

For a reference copy of the document with all sections, see nature.com/documents/nr-reporting-summary-flat.pdf

# Life sciences study design

All studies must disclose on these points even when the disclosure is negative.

| | |
|---|---|
| Sample size | 6245, sample size arrived including participants with 2-doses of mRNA COVID-19 vaccine and anti-S1 measurements in C4R |
| Data exclusions | Excluded participants who did not have 2 doses of mRNA COVID-19 vaccine or did not have valid anti-S1 measurements |
| Replication | Replication was not performed as this was a meta-cohort analysis of epidemiological data. |
| Randomization | Randomization not applicable to this study given this was not a clinical trial or testing an intervention. |
| Blinding | Blinding not applicable given this study was not testing an intervention. |

# Reporting for specific materials, systems and methods

We require information from authors about some types of materials, experimental systems and methods used in many studies. Here, indicate whether each material, system or method listed is relevant to your study. If you are not sure if a list item applies to your research, read the appropriate section before selecting a response.

## Materials & experimental systems

| n/a | Involved in the study | |
|---|---|---|
| ☒ | ☐ | Antibodies |
| ☒ | ☐ | Eukaryotic cell lines |
| ☒ | ☐ | Palaeontology and archaeology |
| ☒ | ☐ | Animals and other organisms |
| ☒ | ☐ | Clinical data |
| ☒ | ☐ | Dual use research of concern |
| ☒ | ☐ | Plants |

## Methods

| n/a | Involved in the study | |
|---|---|---|
| ☒ | ☐ | ChIP-seq |
| ☒ | ☐ | Flow cytometry |
| ☒ | ☐ | MRI-based neuroimaging |

## Plants

| | |
|---|---|
| Seed stocks | *Report on the source of all seed stocks or other plant material used. If applicable, state the seed stock centre and catalogue number. If plant specimens were collected from the field, describe the collection location, date and sampling procedures.* |
| Novel plant genotypes | *Describe the methods by which all novel plant genotypes were produced. This includes those generated by transgenic approaches, gene editing, chemical/radiation-based mutagenesis and hybridization. For transgenic lines, describe the transformation method, the number of independent lines analyzed and the generation upon which experiments were performed. For gene-edited lines, describe the editor used, the endogenous sequence targeted for editing, the targeting guide RNA sequence (if applicable) and how the editor was applied.* |
| Authentication | *Describe any authentication procedures for each seed stock used or novel genotype generated. Describe any experiments used to assess the effect of a mutation and, where applicable, how potential secondary effects (e.g. second site T-DNA insertions, mosiacism, off-target gene editing) were examined.* |
